# Repression of SUMOylation pathway by grass carp reovirus contributes to the upregulation of PKR in an IFN-independent manner

**DOI:** 10.18632/oncotarget.20309

**Published:** 2017-08-17

**Authors:** Fei Yu, Longlong Wang, Hao Wang, Jialu Sheng, Liqun Lu

**Affiliations:** ^1^ National Pathogen Collection Center for Aquatic Animals, Shanghai Ocean University, Shanghai, P. R. China; ^2^ Key Laboratory of Agriculture Ministry for Freshwater Aquatic Genetic Resources, Shanghai Ocean University, Shanghai, P. R. China; ^3^ National Experimental Teaching Demonstration Center for Fishery Sciences, Shanghai Ocean University, Shanghai, P. R. China

**Keywords:** grass carp reovirus, SUMOylation, innate immunity, PKR, interferon

## Abstract

SUMOylation, a post-translational modification, is involved in interaction between hosts and viruses, and participates in diverse cellular processes including inflammatory responses and innate immunity. Here, we investigated the interaction between reovirus infection and the cellular SUMOylation machinery using grass carp reovirus (GCRV) as a model. Full-length cDNAs of grass carp SUMO-1 and SUMO-2 were obtained and phylogenetic analysis indicated that they shared high homology with those of higher vertebrates. The two modifiers and SUMO conjugating enzyme 9 (Ubc9) were ubiquitously expressed in all tested tissues of grass carp. During GCRV infection in CIK cells, transcriptional expressions of SUMO1/2 and Ubc9 were significantly inhibited; while UV-inactivated GCRV failed to inhibit the expression of the three molecules, which suggested that SUMOylation system was suppressed during viral replication. In CIK cells treated with inhibitor 2-D08 for SUMOylation, GCRV replication was not interfered; however, transcriptional analysis of immune genes involved in anti-viral interferon (IFN) response indicated that IRF2 and PKR were significantly up-regulated in CIK cells treated with inhibitor in contrast to IRF1, IRF7 and IFNI. Furthermore, 2-D08 treatment coupled with GCRV challenge resulted in higher IRF2 and PKR level during infection in comparison to those of CIK cells infected with GCRV only. These results indicated that inhibition of SUMOylation should result in the induction of PKR via IFN-independent manner, and both IFN-signaling and IFN-independent signaling seemed to involve in the upregulation of PKR during the process of GCRV infection. Repression of SUMOylation by GCRV might represent a cellular antiviral mechanism.

## INTRODUCTION

SUMOylation plays an important role on the maintenance of life activity and diversity of protein function, and is involved in cancer, neurological disease, heart disease and intrinsic innate immunity [1, 2]. Small ubiquitin-like modifier (SUMO) only shares about 18% homology with ubiquitin modifier and has been found to bind to the lysine residue on the specific consensus sequence of target protein. Similar to the ubiquitin pathway, SUMO isoforms (SUMO-1, SUMO-2/3, SUMO-4) in eukaryotes are conserved small proteins, which are covalently conjugated to substrate proteins by a different set of enzymes: E1 activating enzyme, E2 conjugating enzyme and E3 ligase [3]. SUMO conjugating enzyme 9 (Ubc9) is the sole SUMO E2 enzyme, which is involved in the conjugation of SUMO isoforms to many diverse substrates [4–6].

Grass carp reovirus (GCRV) has served as a protype of aquareoviruses (family Reoviridae, genus Aquareovirus) due to its strong pathogenicity for grass carp *Ctenopharyngodon idellus* by causing grass carp hemorrhagic disease; it has also been extensively studied for the interaction mechanism between dsRNA virus and dsRNA-initiated antiviral response [7]. In our previous study, grass carp Ubc9 bound to the N-terminal coiled-coil domain of GCRV-104 fiber protein and promoted viral infection efficiency [8, 9]. However, it remains to be clarified whether direct SUMOylation of viral target or SUMOylation-mediated innate immune response is responsible for the pro-viral effect of Ubc9. In various virus-cell infection models, strong associations between SUMOylation and inflammatory responses/innate immunity have been reported [2]. For instance, SUMOylation inhibited inflammation by silencing IFN expression and LPS-induced anti-viral response was enhanced by SUMO deficiency in bone marrow cells [10]; SUMOylation of IRF2 regulated its own transcription to inhibit IRF1 activity in 293 cells [11]; Over-expressed SUMO1 and SUMO2 enhanced Singapore grouper iridovirus and red-spotted grouper nervous necrosis virus replication during viral infection *in vitro* [12] and SUMO expression reduced interferon synthesis upon rabies virus or vesicular stomatitis virus infection by protecting MxA protein from degradation [13]. Nevertheless, whether SUMOylation influences innate immunity pathway of bony fish is still not clear.

Interferons (IFNs) are crucial cytokines with pivotal roles in host immunity and first-line innate defense against viral pathogens [14]. Mammalian IFNs include three groups: type I IFNs, type II IFNs, and type III IFNs; while all fish IFNs belong to type I IFN, which is thought to be initiated through the pattern recognition of virus component by Toll-like receptors (TLRs) and Retinoic acid inducible gene 1-like receptors (RLRs) [7]. Signals from TLRs or RLRs are then transmitted to IFN regulatory factors (IRFs) which would translocate from cytoplasm to nucleus to turn on IFN gene transcription by binding to its promoter; and IFN expression induces the expression of many IFN-stimulated genes (ISGs) via JAK-STAT signaling pathway, which include PKR, Mx, PKZ, TRIMs, IFNI et al [7, 15]. In grass carp, transcription of IFNI is reported to be upregulated by GCRV infection, regardless of its efficient replication in target tissues or cell lines [7].

This study aims to monitor the SUMOylation machinery during GCRV infection by quantitatively detecting the transcription levels of SUMO1, SUMO2 and Ubc9, to investigate whether SUMOylation was necessary for GCRV replication in CIK cells through a specific SUMOylation-inhibitor assay, and to probe the effect of regulated SUMOylation level on host antiviral response with a focus on immune genes involved in IFN response. This is a pilot tentative to reveal the host antiviral defense in response to regulated SUMOylation pathway in bony fish, which may provide insights into the post-translational modification control of antiviral host defense.

## RESULTS

### Characterization of grass carp SUMO1 and SUMO2

**Figure 1:**
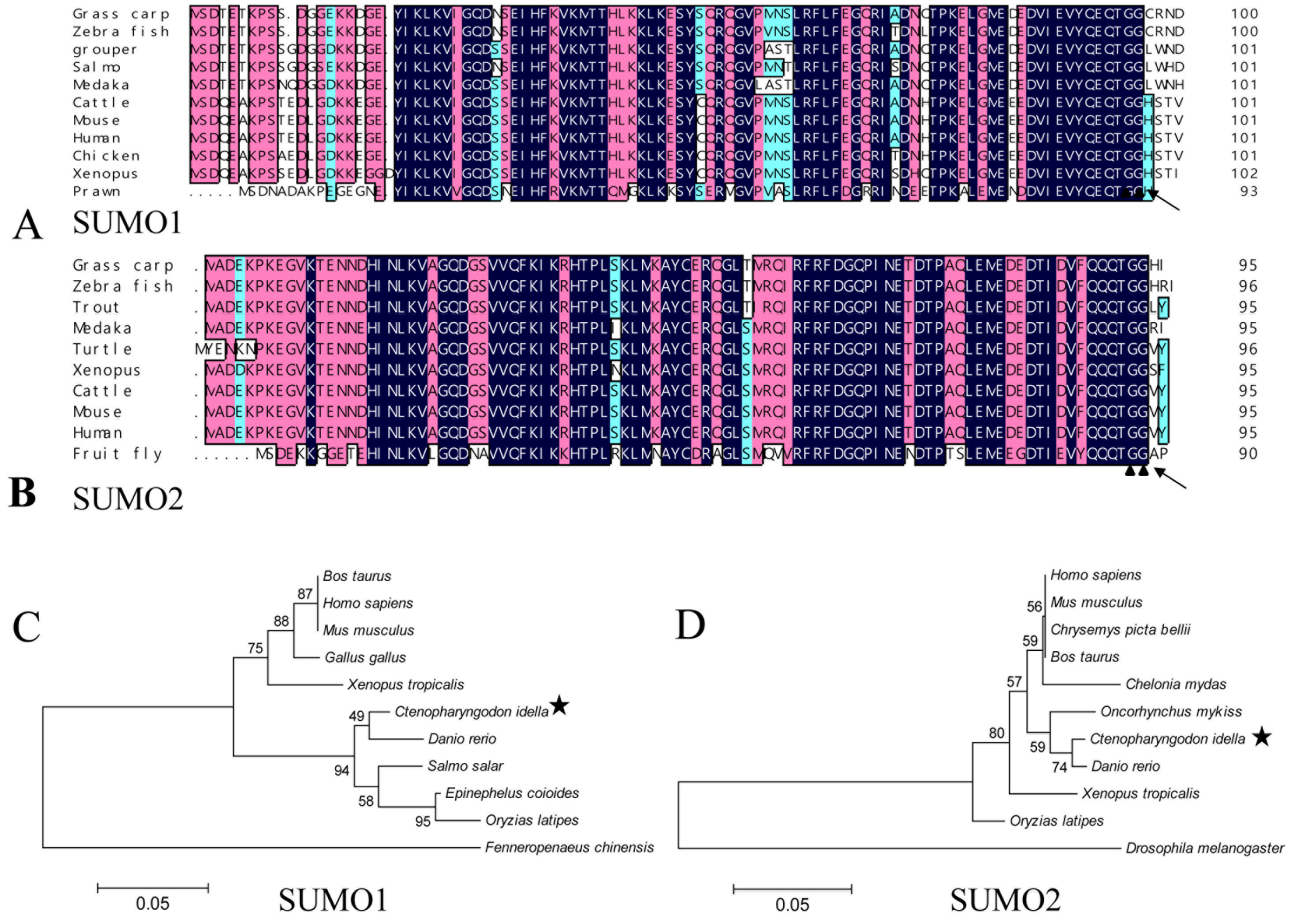
Sequence analysis of grass carp SUMO1 and SUMO2 **A.** and **B.** amino acid sequence multi-alignments of SUMO1 and SUMO2 from different organisms; **C.** and **D.** the phylogenetic analysis between SUMO1/SUMO2 and their homologues from other organisms. In A and B, the blue (100%), pink (above 75%), and cyan (above 50%) bars indicate the similarity at a specific position; the particular double-Gly motifs are shown by solid triangles; and the predictive cleavage sites are marked using black arrows in the SUMO sequences. Species and accession numbers for SUMO1 proteins: Grass carp [MF106225]; Zebra fish [NM_213159.1]; Grouper [ALR35733.1]; Salmo [BT058601.1]; Medaka [GQ463435.1]; Cattle [AAX09006.1]; Mouse [NP_033486.1]; Human [NP_003343.1];Chicken [NP_989466.1]; Xenopus [Q6DEP7.1]; Prawn [AHE40942.1]; Danio rerio [NM_213159.1]; Bos Taurus [AAX09006.1]; Ctenopharyngodon Idella [MF106225]; Epinephelus coioides [ALR35733.1]; Fenneropenaeus chinensis [AHE40942.1]; Gallus gallus [NP_989466.1]; Homo sapiens [NP_003343.1]; Mus musculus [NP_033486.1]; Oryzias latipes [GQ463435.1]; Salmo salar [BT058601.1]; Xenopus tropicalis Q6DEP7.1. Species and accession numbers for SUMO2 proteins: Grass carp [MF106226]; Zebra fish [NP_001003422.1]; Trout [NP_001158529.1]; Medaka [NP_001165519.1]; Turtle [XP_007065106.1]; Xenopus [NP_001016406.1]; Cattle [NP_777194.1]; Mouse [NP_579932.1]; Human [NM_006937.3]; Fruit fly [NP_477411.1]; Danio rerio [NP_001003422.1]; Ctenopharyngodon Idella [MF106226]; Oncorhynchus mykiss [NP_001158529.1]; Drosophila melanogaster [NP_477411.1]; Chrysemys picta bellii [XP_005297527.1]; Homo sapiens [NM_006937.3]; Mus musculus [NP_579932.1]; Oryzias latipes NP_001165519.1]; Bos Taurus [NP_777194.1; Xenopus tropicalis [NP_001016406.1]; Chelonia mydas [XP_007065106.1].

Taking advantage of the transcriptome data of grass carp achieved by RNA-seq [16], the partial coding sequence of SUMO 1 and SUMO2 were identified, and the two complete ORFs were amplified and assembled according to the known sequences from cDNA template by PCR. The full-length cDNA of the SUMO1 gene contains a putative ORF of 303 bp in length followed by 3′ untranslated region including a typical poly adenylation signal (GenBank accession number: MF106225). The molecular mass of SUMO1 protein (100 amino acids, aa) is about 11412 Da, and its predicted isoelectric point is 5.18. The nearly full-length cDNA of the SUMO2 gene with a putative ORF of 288 bp in length (GenBank accession number: MF106226) encoding 95 amino acids. The deduced molecular mass of SUMO2 protein is 10873 Da, with an isoelectric point of 5.55. The predictive amino acid sequences of grass carp SUMO1 and SUMO2 were shown and aligned with other SUMOs of the indicated species (Figure 1A and 1B). SUMOs are cleaved by sentrin-specific proteases (SENPs) to expose the C-terminal double-Gly motif before adenylation [17]. Both grass carp SUMO1 and SUMO2 possess a double-Gly motif at C-terminus, which is an active site of the covalent binding to the substrate. The cleavage sites of grass carp SUMOs also locate at the C-terminus. The phylogenetic trees between SUMO1/SUMO2 and their homologues from other species were generated based on amino acid sequences (Figure 1C and 1D), and the results showed that SUMO1 and SUMO2 proteins shared highly identities with those of other vertebrates, while less identity to those of the arthropod.

**Figure 2:**
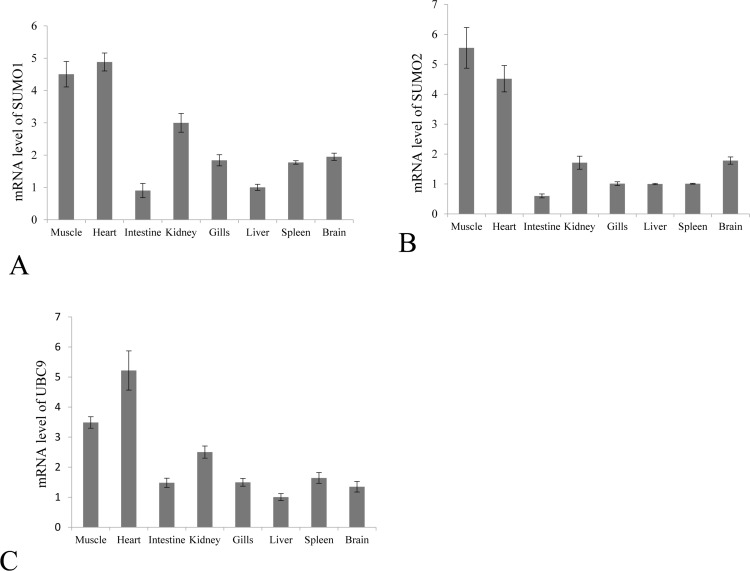
Constitutive tissue distribution of SUMO1, SUMO2 and Ubc9 in grass carp The mRNA levels of SUMO1 **A.** SUMO2 **B.** and Ubc9 **C.** were measured by real time RT-PCR in the muscle, heart, intestine, kidney, gill, liver, spleen and brain. The relative expression levels of SUMO1, SUMO2 and Ubc9 were normalized to 18S rRNA level and calculated against the expression level in the liver using the 2 -ΔΔCT method. Each tissue was sampled three times from three fish, and fold change are presented as the mean ± SE (*n* = 3).

SUMO1/2 and Ubc9 are among the key molecules in host SUMOylation machinery, and we have reported the involvement of Ubc9 in the interaction between grass carp reovirus and host cells [8]. The transcription levels of Grass carp SUMO1/SUMO2 and Ubc9 in muscle, heart, intestine, kidney, gill, liver, spleen and brain were quantitatively measured by real time RT-PCR (Figure 2). As expected, grass carp SUMO1, SUMO2 and Ubc9 were ubiquitously expressed in all the analyzed tissues in healthy grass carp, which was in consistence with the fact that SUMOylation played a primary role on post-translational modification in cellular life [5].

### Transcriptional repression of SUMO1, SUMO2 and Ubc9 by GCRV

**Figure 3:**
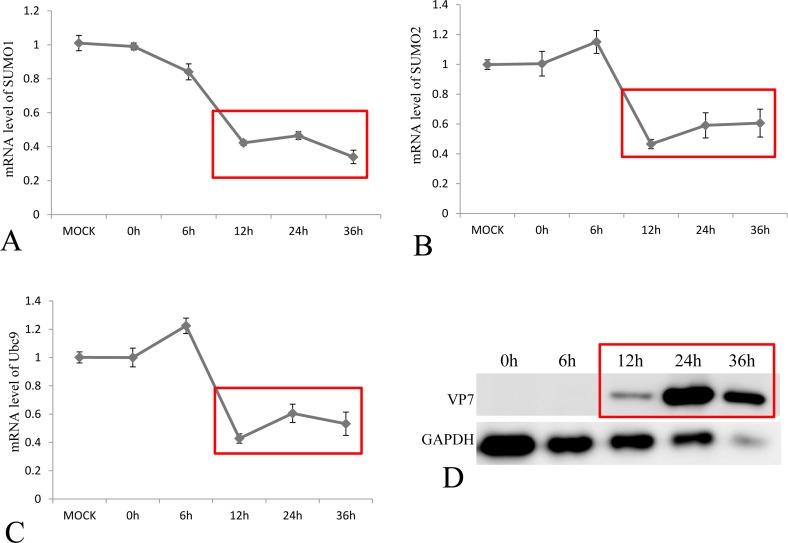
Transcriptional repression of SUMO1, SUMO2 and Ubc9 by GCRV in CIK cells The CIK cells were infected by GCRV-JX01 at a MOI of 0.1, and the relative mRNA levels of SUMO1 **A.** SUMO2 **B.** and Ubc9 **C.** were determined by real time RT-PCR and normalized to 18S rRNA level and calculated against the expression levels of MOCK group by the 2 -ΔΔCT method. The protein expression of GCRV VP7 was probed by anti-VP7 polyclonal antibody and the GAPDH protein acted as internal control **D.** The process of viral replication was shown by red rectangles and the time points for sample collection were indicated.

**Figure 4:**
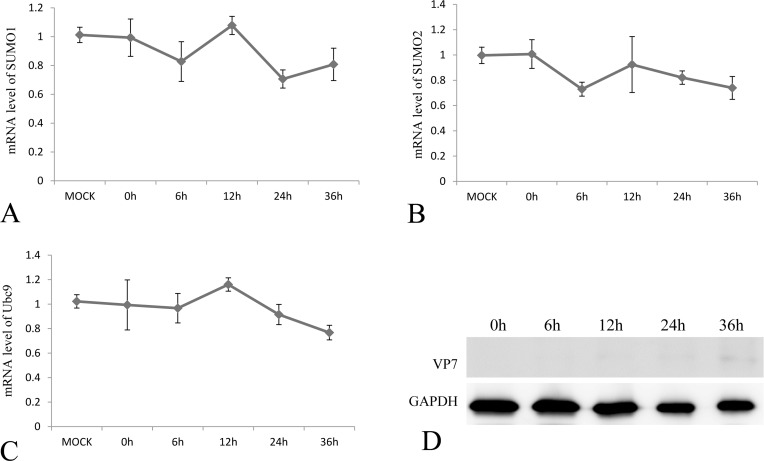
The mRNA expression levels of SUMO1, SUMO2 and Ubc9 in CIK cells challenged with UV-inactivation GCRV-JX01 particles The CIK cells were infected by 0.1 MOI GCRV (Inactivated by UV), and the relative mRNA levels of SUMO1 **A.** SUMO2 **B.** and Ubc9 **C.** were determined by real time RT-PCR and normalized to 18S rRNA level and calculated by comparing to the expression levels of MOCK group by the 2 -ΔΔCT method. The protein expression of VP7 post infection was probed by anti-VP7 polyclonal antibody and the GAPDH protein acted as internal control **D.** The time points for sample collection were indicated.

To investigate the effect of GCRV infection on the SUMOylation pathway, transcriptional steady state levels of SUMO1, SUMO2 and Ubc9 were determined by real time RT-PCR (Figure 3A, and 3C) during the infection course of GCRV, which was monitored by probing the expression of outer capsid protein VP7 in infected CIK cells (Figure 3D). The results demonstrated that the expressions of SUMO1, SUMO2 and Ubc9 were significantly inhibited at 12 h, 24 h and 36 h post infection, which correlated with robust expression of viral protein VP7 reflecting GCRV replication. The result suggested that active viral replication might be responsible for the repression of these molecules. To further prove the hypothesis, GCRV particles were inactivated by ultraviolet and subjected to infect CIK cells (Figure 4D). Transcriptional analysis of the SUMOs and Ubc9 indicated that inactivated GCRV induced no repression of SUMO1, SUMO2 and Ubc9 in comparison to those of mock-infected cells (Figure 4A, and 4C). Thus, host SUMOylation machinery seemed to be systematically repressed during the process of GCRV replication.

### Replication of GCRV independent of active SUMOylation

**Figure 5:**
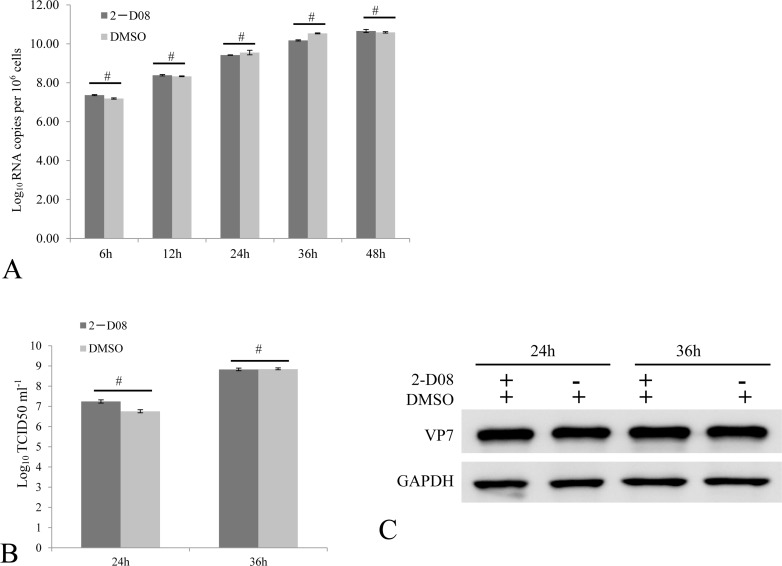
Active SUMOylation is not required for efficient GCRV replication The CIK cells treated with 100 uM 2-D08 were infected by 0.01 MOI GCRV. The relative fold changes of viral S10 mRNA were quantitatively measured by real time RT-PCR to reflect viral replication level at the indicated time points post infection **A.** The viral titer of cell supernatant **B.** by a TCID_50_ assay, as well as the cellular viral protein level by immunoblotting assay **C.** were monitored at 24 and 36 h post infection. The VP7 protein expression of cells was determined by anti-VP7 polyclonal antibody and the GAPDH protein acted as internal control. DMSO treated cells served as negative control in these assays. No significant differences were marked by number sign (#).

What is the reason for the viral repression of host SUMOylation machinery? One reasonable explanation might be that SUMOylation of viral proteins served as an antiviral approach for host cells, and GCRV had to antagonize SUMOylation for efficient viral replication. To clarify this, we employed the specific inhibitor 2-D08 of SUMOylation to determine whether inhibition of active SUMOylation was necessary for efficient replication of GCRV. At 6, 12, 24, 36 and 48 h post GCRV infection, the viral mRNA of S10 fragment in challenged cells treated with or without 2-D08 was measured, which demonstrated no difference in the level of viral S10 at each tested time points (Figure 5A). At the time point of 24 and 36 h post challenge, tissue culture supernatants were harvested for TCID_50_ analysis of GCRV progeny (Figure 5B), and infected cells were collected for immunoblotting analysis of viral capsid protein VP7 (Figure 5C). Figure 5B and 5C indicated that no detectable difference in viral progeny production existed between 2-D08 treated cells and the control. Thus, inhibition of SUMOylation was not required for efficient viral replication *in vitro*. In consistence with this, GCRV structural proteins (VP1/ VP2/ VP3/ VP4/ VP5/ VP6/ VP7) were determined to be not potential interacting partners for grass carp SUMOs, which was revealed by a systematic yeast two-hybrid screening (Supplementary Figure 1).

### Stimulation of IRF2 and PKR through inhibition of SUMOylation

**Figure 6:**
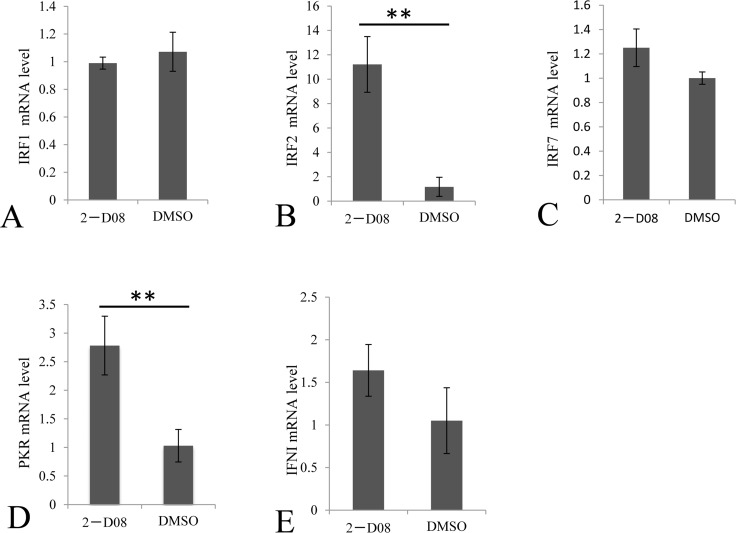
Regulation of selected immune genes by SUMOylation inhibitor in CIK cells The CIK cells were treated with 100 μM 2-D08 and mock cells were treated with equivalent DMSO. The relative mRNA expression levels of IRFI **A.** IRF2 **B.** IRF7 **C.** PKR **D.** and IFNI **E.** were measured by real time RT-PCR and normalized to 18S rRNA level and calculated by comparing to the expression levels of the mock cells by the 2 -ΔΔCT method. Significant differences were considered at * *p* < 0.05.

If replication of GCRV does not benefit from the repression of SUMOylation, it's reasonable to speculate that repressed SUMOylation in infected cells might result from host defense to regulate cytokine expressions to active antiviral responses in infected cells or surrounding cells. To prove this hypothesis, we tested the transcriptional levels of selected cytokines involved in the IFN signaling in cells treated W/O 2-D08 (with or without 2-D08), which was known to function through blocking the transfer of SUMO from Ubc9 thioester conjugate to the substrate. In the SUMOylation inhibition assay, the IFR2 and PKR genes were induced to a significantly higher level in 2-D08 treated cells (Figure 6B and 6D), and the mRNA levels of IRF1, IRF7 and IFNI were not statistically altered by De-SUMOylation (Figure 6A, and 6E) comparing to the mock CIK cells. Interestingly, IRF2 is a bifunctional transcriptional regulator, which acts as an inhibitory factor of interferon signaling genes and a positive regulatory gene for IL-7 and Gig2 [18, 19]. Thus, induced IFR2 predicted an inhibited IFN response, which correlated with the statistically constant level of IFNI in cells treated with SUMOylation inhibitor in comparison to that of normal cells. Our data indicated that induction of PKR by SUMOylation inhibitor should be resulted from a pathway independent of IFNI activation.

### Involvement of IFN signaling and IFN-independent signaling in inducing PKR by GCRV

**Figure 7:**
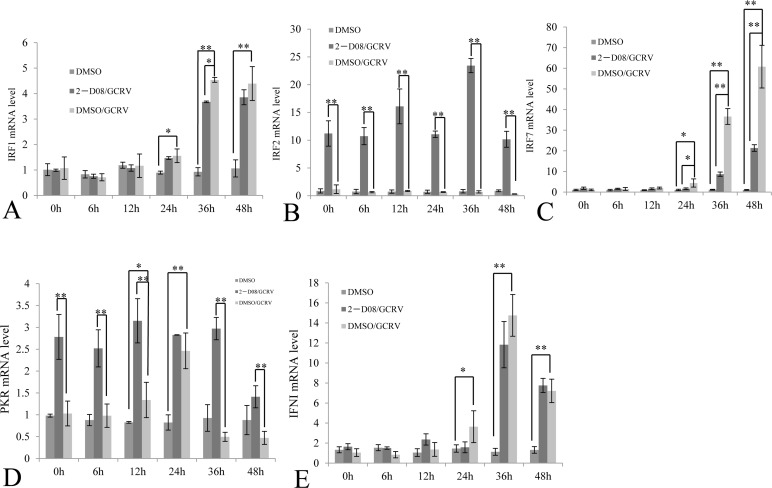
Comparative analysis of selected immune gene expression between GCRV-challenged cells and those treated with both GCRV and 2-D08 The CIK cells treated with 100μM 2-D08 and mock cells treated with equivalent DMSO; CIK cells were infected by 0.01 MOI GCRV. The expressions of IRFI, IRF2, IRF7, PKR and IFNI were quantitatively measured post infection by GCRV-JX01 in CIK cells treated w/o SUMOylation inhibitor 2-D08, while DMSO-treated CIK cells served as a control. The relative mRNA expression levels of IRFI **A.** IRF2 **B.** IRF7 **C.** PKR **D.** and IFNI **E.** were measured by real time RT-PCR and normalized to 18S rRNA level and calculated by comparing to the expression levels of the mock cells at 0 h by the 2 -ΔΔCT method. Significant differences were considered at * p < 0.05 and highly significant at ** *p* < 0.01.

IFN and PKR had been reported to be upregulated in response to GCRV infection [7], which suggested that IFN-transcriptional inhibitor IRF2 shouldn't be over-expressed during viral infection. To confirm this, we monitored the steady-state levels of IRF2 during the course of GCRV infection (Figure 7B), as well as IRF1 (Figure 7A), IRF7 (Figure 7C), PKR (Figure 7D) and IFNI (Figure 7E). Healthy cells treated with DMSO only served as negative control, while cells treated with both 2-D08 and GCRV were employed for evaluation of the synergetic effect in regulating IFN signaling genes between inhibitor of SUMOylation and GCRV replication. The results showed that transcriptional activators IRF1 and IRF7 were statistically upregulated as early as 24 h post infection in cells W/O 2-D08 treatment (Figure 7A and 7C), as well as their activating target IFNI (Figure 7E); in contrast, GCRV infection induced no expression of IFN-transcriptional inhibitor IRF2, which could only be significantly stimulated in cells treated with both 2-D08 and GCRV (Figure 7B) or 2-D08 only (Figure 6B). Compared with normal cells treated with 2-D08 only (Figure 6), GCRV infection coupled W/O 2-D08 treatment (Figure 7) tent to systematically activate IFN signaling by inducing IRF1, IRF7 and IFN, and refrained from expression of IRF2 to facilitate the stimulation of IFN signaling. The data indicated that GCRV infection dominated SUMOylation in regulating IFN signaling with mechanisms not fully understood yet.

It's interesting to document the upregulating nature of PKR in cells treated with 2-D08 (2.8 folds in Figure 6D), cells infected with GCRV (1.5 folds at 12 h p.i. and 2.5 folds at 24 h p.i. in Figure 7D), and cells treated with both 2-D08 and GCRV (3.1 folds at 12 h p.i, and 2.8 folds at 24 h p.i in Figure 7D). Overall, GCRV infection seemed to pose no dominant impact on the stimulated level of PKR in comparison to the inducing efficiency of SUMOylation inhibitor 2-D08. As a typical ISG, PKR was known to be upregulated by IFN; however, as early as 12 h p.i., PKR was statistically stimulated while no significant induction of IRF1, IRF7, or IFN was detected at that time point (Figure 7A, and 7E). Thus, IFN-independent signaling should be significantly involved in the stimulation of PKR, especially during the early phase of viral infection. Although it was impossible to differentiate the contribution of IFN signaling from IFN-independent signaling in upregulating PKR during the late infection of GCRV (Figure 7D), IFN-signaling activated by GCRV challenge did result in a weaker stimulation efficiency of PKR than IFN-independent signaling induced by 2-D08 treatment in our quantitative assays.

## DISCUSSION

In the process of evolution, host cells have utilized SUMOylation pathway, a specific post-translational modification, to enhance possible defense pathways against invading pathogens or to maintain cellular homeostasis under stress [11]. Four SUMO paralogs in human have been identified, among which SUMO2, 3 and 4 share high homology with each other, and SUMO1, 2, and 3 can act as protein modifiers, whereas SUMO4 seems to be expressed only in limited tissues and may have no ability to be conjugated to substrate proteins [20, 21]. Grass carp SUMO1 and SUMO2 identified here shared low homology with each other (Supplementary Figure 2) and they should be classified into two subfamilies; and the both hold high identities with their homologues in other organisms, which predicted the conservative functions of the SUMOylation pathway (Figures 1 and 2).

However, viruses have evolved elaborate means to modulate sumoylation in facilitating viral replication or to take advantage of the post-translational process in regulating virus-encoded protein function [3]. Furthermore, modulation of sumoylation levels had been implicated in evasion of host immune response by pathogens including bacteria, parasites and viruses [1, 22]. Thus, it's not surprising to reveal that grass carp infection results in significant suppression of SUMO1, SUMO2 and Ubc9, which constitute the key molecules in SUMOylation machinery (Figures 3 and 4). Previously, we have demonstrated that overexpression of Ubc9 resulted in increased viral replication efficiency and Ubc9 might act as a proviral factor. Thus, repression of SUMOylation machinery seemed to constitute a functional host antiviral response. Is the suppression of SUMOylation preferred by GCRV? The answer is “No”. Taking advantage of the specific inhibitor of SUMOylation, 2-D08, GCRV replicated efficiently in cells with blocked SUMOylation (Figure 5). SUMOylation is important for the regulation of many cellular proteins and pathways [1], and the antiviral IFN-response was chosen here to investigate the consequence of suppressed SUMOylation in grass carp, which was largely due to the fact that it served as the first line in antiviral immune response for all organisms.

IFN had been reported to regulate over 500 ISGs. Among them, PKR functions to shut down viral protein translation through phosphorylation of Ser51 of the alpha subunit of eIF2 as a defense mechanism against viral infection [23]. In contrast to the IFN-inhibitory regulator IRF2 [11, 19], IRF1, IRF3 and IRF7 are three multifunctional and critical transcription factors essential for the transcription of type 1 IFN [24, 25]. IRF3 and IRF7 belonged to the IRF3 subfamily and they cooperatively regulated the expression of IFN [26]. In this study, IRF2, IRF1, and IRF7 were selected to monitor the expression of interferon regulatory factors; while IFNI was picked for evaluation of IFN response with PKR as a marker for ISGs expression (Figures 6 and 7). One of the most exciting discovery lied in that IRF2 was dramatically induced by 2-D08, which predicted a non-IFN signaling response for the induced PKR (Figure 6). Consistently, IFNI, IRF1, and IRF7 were not stimulated in cells treated with 2-D08, which further validated the existence of non-IFN signaling (Figure 6). It was worth to note that ISGs were generally regulated by many regulators besides IFN, for example, RIG-I had been identified as a key antiviral interferon-stimulated gene against hepatitis E virus regardless of interferon production [27].

The cellular cytokine network is generally finely regulated by different mechanisms and components. Upon GCRV challenge, we demonstrated that IRF1, IRF7, IFN and PKR were ubiquitously induced in contrast to IRF2 (Figure 7), which was in consistence with previous reports [7]. GCRV challenge coupled with 2-D08 treatment did recover the induction of IRF2, which suggested that complete abolishment of SUMOylation is required for efficient stimulation of IRF2. PKR was induced in cells treated with either of 2-D08, GCRV or both of them (Figures 5, 6, and 7), and GCRV challenge did result in a weaker stimulation efficiency of PKR (less than 2.5 folds) than 2-D08 treatment through IFN-independent signaling (2.8 folds in Figure 7). Thus, IFN-independent signaling should participate in the upregulation of PKR, especially during early infection (12 h p.i.) when PKR was statistically induced in contrast to IFN, IRF1, and IRF7 (Figure 7).

In summary, significant suppression of SUMOs and Ubc9 genes by grass carp reovirus was revealed in this study, and replication of grass carp reovirus was demonstrated to be independent of the inhibition of cellular SUMOylation machinery. Furthermore, 2-D08, inhibitor of SUMOylation, was shown to upregulate PKR through an IFN-independent manner, and both IFN signaling and IFN-independent signaling were involved in induction of PKR during GCRV infection.

## MATERIALS AND METHODS

### Cell line and virus

*Ctenopharyngodon idellus* kidney (CIK) cell line was obtained from China Center for Type Culture Collection (CCTCC, Wuhan) and maintained in M199 medium supplemented with 10% fetal bovine serum (Gibco, USA) at 28°C. GCRV-JX01 was isolated from Jiangxi province, and propagated by infecting CIK cells [28]. The viral titer was measured by the TCID50 method and the GCRV-JX01 was inactivated by ultraviolet (UV) as previously described [29].

### RNA extraction and cDNA synthesis

Healthy grass carps (200-300 g) were obtain from an offspring farm in Suzhou City, Jiangsu province, China. All tested tissues were sampled from 3 grass carps. Total RNA was extracted from 80 mg tissue or cell samples (1×10^6^ cells) using 1 ml of TRIzol (Invitrigen, USA) according to the guide instruction. 200 ng of isolated RNA were reverse transcribed into cDNA using PrimeScript™ 1st Strand cDNA Synthesis Kit (TaKaRa, Janpan) for gene cloning or PrimeScript™ RT reagent Kit for real time PCR analysis.

### Molecular cloning and bioinformatic analysis

The cDNA of SUMO1 and SUMO2 were amplified from cDNA of grass carp kidney tissue by RT-PCR, and the primers (detailed in Table 1) were designed based on the transcriptome data [16] and cDNA library constructed for the yeast two-hybrid screening [30].The rapid amplification of cDNA ends was carried out using the SMARTer_RACE cDNA Amplification kit (Clontech). The DNA fragments of SUMO1 and SUMO2 were cloned into pMD19-T vector (Takara, Japan) and sequenced (Shangon, China). The mRNA sequences of SUMO1 and 2 have been deposited to GeneBank with the accession No. of MF106225 and MF106226, respectively. The homologous sequences of SUMO1and SUMO2 of other vertebrate organisms were obtained from Genbank database (NCBI, https://www.ncbi.nlm.nih.gov/). The coding sequence (CDS) was predicted by DNAssit 2.1 software. The protein molecular weight and isoelectric point were deduced using the software from website (http://web.expasy.org/compute_pi/). Based on the amino acid sequences from other species, DNAman 4.0 software was used to produce multiple alignments and the MEGA version 5.1 was used to conduct phylogenetic trees by neighbor-joining (NJ) method.

**Table 1:** Primers used in this study

Gene	Forward Primer 1(5′-3′)	Reverse Primer (5′-3′)	Aplication
SUMO-1	AAGTGATGGAGGCGAGAAGA	AGAAGGCAGGGATTGGTTAG	Cloning
SUMO-1	TAATGTCAGATACGGAGACCAAG	TATCCAAGACCAGGCAGAATAGG	Cloning
SUMO-2	AGAACAACGACCACATCAACCTG	TCATATCTAAACCCGAGCGAAAC	Cloning
SUMO-1	AGTGATCGGTCAGGACAACAG	ATCTTCCATTCCCAGCTCTTT	Real time RT-PCR
SUMO-2	GAACAACGACCACATCAACCT	CTTCATCCTCCATTTCCAACT	Real time RT-PCR
UBC9	TTATGAACTGGGAATGTGC	CTTTGGAGGTGATGAGGG	Real time RT-PCR
IRF1	TCATTGAGATTTCACGGCA	CAGAGAGGACACATGGTCG	Real time RT-PCR
IFR2	TACAGAGGCTGATGGGCGGA	GAGGGGGGACGAGGGGAAAG	Real time RT-PCR
IRF7	GAAGAGACCTTGGGGACGAG	TTGAGGACGGATAATGCGAT	Real time RT-PCR
PKR	ACTAAAAGGACAGGAACACG	TTCAGGACTGGGACTCAACA	Real time RT-PCR
IFN	CATTGCCAACAGACGATA	ATTAGCTTGCTTGATCAGATT	Real time RT-PCR
18S rRAN	ATTTCCGACACGGAGAGG	CATGGGTTTAGGATACGCTC	Real time RT-PCR
GCRV-S10	CAAGACCATTCAAGACTC	TCACTCACTTCGACTAAT	Real time RT-PCR

### Cell treatment by 2-D08 and sample collection

2-D08 (2′, 3′, 4′-trihydroxyflavone, Sigma) was a cell-permeable inhibitor of SUMOylation that block the transfer of SUMO from Ubc9 thioester conjugate to the substrate [31]. SUMOylation could be inhibited in cells by 100 μM 2-D08 [31, 32]. The M199 medium was supplemented with 10% fetal bovine serum and 100 μM 2-D08. 1× 10^6^ CIK cells per well was cultured for 24 h in 6-well plates, then the cells was subjected directly for analysis of signals involved in IFN pathway, or challenged by 0.1 or 0.01 TCID_50_ GCRV-JX01 before transcriptional analysis. Samples were collected at different time points post treatments.

### Real time RT-PCR

The primers of all listed genes used for real time RT-PCR were shown in Table 1. The expression levels of SUMO1, SUMO2 and Ubc9 in tissues of grass carp or in CIK cells were determined in triplicates by real time RT-PCR and normalized to the 18S rRNA level. Real time RT-PCR was performed according to the SYBR^®^ Premix Ex Taq II (TaKaRa, Japan) kit instructions. The real time RT-PCR conditions were as follows: 95°C for 10 min, then 40 cycles of 95°C for 10 s, 52-60°C for 15 s, and 72°C for 20 s. The relative fold changes were calculated by comparing to the corresponding controls. GCRV-S10 mRNA level was used to evaluate the viral transcription levels in the viral infection assay [28]. Transcriptional analysis of selected immune genes involved in IFN response was performed by Real time RT-PCR assay as described above, which included IRF1 (GU997098.1), IRF2 (JX628585), IRF7 (GQ141741.1) PKR(JX511974.1), and IFNI (GU139255.1).

### Immunoblotting assay

Immunoblotting assay was performed as described previously. Briefly, the protein samples were resolved by 10% or 12% SDS-PAGE and then transferred onto 0.45 μm polyvinylidene fluoride (PVDF) membrane (Merck millipore, Germany), then the membrane were blocked for 2 h at room temperature in 5% non-fat milk dissolved in PBST (140 mM NaCl, 2.5 mM KCl, 10 mM Na_2_HPO4, 2 mM KH_2_PO4 and 0.1% Tween-20). The homemade primary antibody (anti-VP7, 1:4000) and secondary antibody (HRP conjugated anti-mouse IgG, 1:5000, Abmart, China) were used to probe the target protein. Expression of GAPDH (anti-GAPDH,1:4000, Abclonal, China) was used as an internal control. Bands were visualized using chemiluminescence kit (Thermo Fisher, USA).

### Statistical analysis

Data analysis was performed using SPASS software and significant differences were considered at p < 0.05 and highly significant at p < 0.01.

## SUPPLEMENTARY MATERIALS FIGURES AND TABLES



## Abbreviations

GCRV: Grass Carp Reovirus
dsRNA: Double Stranded RNA
SUMO: Small Ubiquitin-like Modifier
Ubc9: SUMO Conjugating Enzyme 9
IRFs: Interferon Regulatory Factors
PKR: dsRNA-activated protein kinase
IFN: Interferon
