# Supplementary material for: Repression of SUMOylation pathway by grass carp reovirus contributes to the upregulation of PKR in an IFN-independent manner
Source: Oncotarget. 2017 Aug 17;8(42):71500–11. doi: 10.18632/oncotarget.20309 (PMC5641065; doi:10.18632/oncotarget.20309)
Supplement: Supplementary file 1 [file oncotarget-08-71500-s001.pdf]

Repression of SUMOylation pathway by grass carp reovirus contributes to the upregulation of PKR in an IFN-independent manner

SUPPLEMENTARY MATERIALS

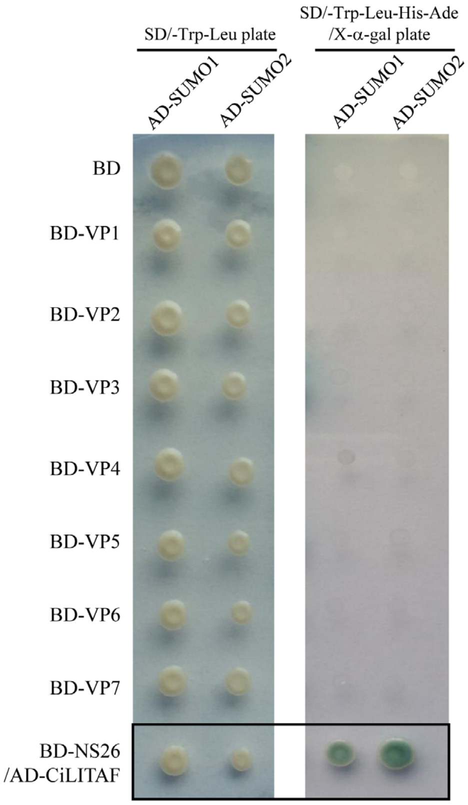

Figure S1. The interaction between grass carp SUMOs and GCRV structural proteins by Yeast-two-Hybrid assay. The bait plasmids pGBKT7 (BD or BD-VP1/VP2/VP3/VP4/VP5/VP6/VP7) and the prey plasmids pGADT7 (AD-SUMO1or AD-SUMO2) were co-transfected into yeast AH109 and indicated. The irrelevant plasmids pGBKT7-NS26 and pGADT7-CiLITAF (BD-NS26 and AD-CiLITAF) were used as a control and the representative colonies were marked by a black rectangle. The yeast transformants were grown on SD/-Trp-Leu plates (Left) and SD/-Trp-Leu-His-Ade/X-α-gal plates (Right). The cyan colonies indicate an interaction between the bait and prey.

```
SUMO1        MSDTETKPSSDGGEKK...DGEYIKLKVIGQDNSEIHFKVKMTTHLKKLKESYSQRQGVP     57
SUMO2        MADEKPKE....GVKTENND..HINLKVAGQDGSVVQFKIKRHTPLSKLMKAYCERQGLT     54

SUMO1        MNSLRFLFEGQRIADNQTPKELGMEDEDVIEVYQEQTGGCRND                     100
SUMO2        MRQIRFRFDGQPINETDTPAQLEMEDEDTIDVFQQQTGGHI                        95
```

Figure S2. Amino acid sequence alignment of grass carp SUMO1 and SUMO2. The blue (100%) and cyan (low or 0%) bars indicate the similarity at a specific position.
